# LONG-TERM CARE FACILITY VARIATION IN THE INCIDENCE OF PNEUMONIA AND INFLUENZA HOSPITALIZATIONS

**DOI:** 10.1093/geroni/igz038.3029

**Published:** 2019-11-08

**Authors:** Elliott Bosco, Andrew Zullo, Kevin McConeghy, Patience Moyo, Robertus van Aalst, Ayman Chit, Vincent Mor, Stefan Gravenstein

**Affiliations:** 1 Brown University School of Public Health, Providence, Rhode Island, United States; 2 Center of Innovation and Long-Term Services and Support, Providence VA Medical Center, Providence, Rhode Island, United States; 3 Sanofi Pasteur, Swiftwater, Pennsylvania, United States

## Abstract

Pneumonia and influenza (P&I) increase morbidity and mortality among older adults, especially those residing in long-term care facilities (LTCFs). Facility-level characteristics may affect P&I risk beyond resident-level determinants. However, the relationship between facility characteristics and P&I is poorly understood. We therefore identified potentially modifiable facility-level characteristics that might influence the incidence of P&I across LTCFs. We conducted a retrospective cohort study using 100% of 2013-2015 Medicare claims linked to Minimum Data Set 3.0 and LTCF-level data. Short-stay (<100 days) and long-stay (≥100 days) LTCF residents aged ≥65 were followed for the first occurrence of hospitalization, LTCF discharge, Medicare disenrollment, or death. We calculated LTCF risk-standardized incidence rates (RSIRs) per 100 person-years for P&I hospitalizations by adjusting for over 30 resident-level demographic and clinical covariates using hierarchical logistic regression. The final study cohorts included 1,767,241 short-stay (13,683 LTCFs) and 922,863 long-stay residents (14,495 LTCFs). LTCFs with lower RSIRs had more Physician Extenders (Nurse Practitioners or Physician’s Assistants) among short-stay (44.9% vs. 41.6%, p<0.001) and long-stay residents (47.4% vs. 37.9%, p<0.001), higher Registered Nurse hours/resident/day among short-stay and long-stay residents (Mean (SD): 0.5 (0.7) vs. 0.4 (0.4), p<0.001), and fewer residents prescribed antipsychotics among short-stay (21.4% (11.6) vs. 23.6% (13.2), p<0.001) and long-stay residents (22.2% (14.3) vs. 25.5% (15.0), p<0.001). LTCF characteristics may play an important role in preventing P&I hospitalizations. Hiring more Registered Nurses and Physician Extenders, increasing staffing hours, and reducing antipsychotic use may be modifiable means of reducing P&I in LTCFs. Funding provided by Sanofi Pasteur.

